# Decreased APOE-containing HDL subfractions and cholesterol efflux capacity of serum in mice lacking *Pcsk9*

**DOI:** 10.1186/1476-511X-12-112

**Published:** 2013-07-24

**Authors:** Seungbum Choi, Aleksandra Aljakna, Ujala Srivastava, Blake R Peterson, Bin Deng, Annik Prat, Ron Korstanje

**Affiliations:** 1 The Jackson Laboratory, Bar Harbor, ME USA; 2 Graduate School of Biomedical Sciences, University of Maine, Orono, ME USA; 3 Department of Medicinal Chemistry, University of Kansas, Lawrence, KS USA; 4 Department of Biology/VGN Proteomics Facility, University of Vermont, Burlington, VT USA; 5 Laboratory of Biochemical Neuroendocrinology, Institut de Recherches Cliniques de Montréal, affiliated to the University of Montreal, Montreal, QC Canada

**Keywords:** Apolipoprotein E, Atherosclerotic fatty streak, Low-density lipoprotein receptor, Macrophage foam cell, Proprotein convertase subtilisin/kexin type 9

## Abstract

**Background:**

Studies in animals showed that PCSK9 is involved in HDL metabolism. We investigated the molecular mechanism by which PCSK9 regulates HDL cholesterol concentration and also whether *Pcsk9* inactivation might affect cholesterol efflux capacity of serum and atherosclerotic fatty streak volume.

**Methods:**

Mass spectrometry and western blot were used to analyze the level of apolipoprotein E (APOE) and A1 (APOA1). A mouse model overexpressing human LDLR was used to test the effect of high levels of liver LDLR on the concentration of HDL cholesterol and APOE-containing HDL subfractions. *Pcsk9* knockout males lacking LDLR and APOE were used to test whether LDLR and APOE are necessary for PCSK9-mediated HDL cholesterol regulation. We also investigated the effects of *Pcsk9* inactivation on cholesterol efflux capacity of serum using THP-1 and J774.A1 macrophage foam cells and atherosclerotic fatty streak volume in the aortic sinus of *Pcsk9* knockout males fed an atherogenic diet.

**Results:**

APOE and APOA1 were reduced in the same HDL subfractions of *Pcsk9* knockout and human LDLR transgenic male mice. In *Pcsk9/Ldlr* double-knockout mice, HDL cholesterol concentration was lower than in *Ldlr* knockout mice and higher than in wild-type controls. In *Pcsk9/Apoe* double-knockout mice, HDL cholesterol concentration was similar to that of *Apoe* knockout males. In *Pcsk9* knockout males, THP-1 macrophage cholesterol efflux capacity of serum was reduced and the fatty streak lesion volume was similar to wild-type controls.

**Conclusions:**

In mice, LDLR and APOE are important factors for PCSK9-mediated HDL regulation. Our data suggest that, although LDLR plays a major role in PCSK9-mediated regulation of HDL cholesterol concentration, it is not the only mechanism and that, regardless of mechanism, APOE is essential. *Pcsk9* inactivation decreases the HDL cholesterol concentration and cholesterol efflux capacity in serum, but does not increase atherosclerotic fatty streak volume.

## Background

*PCSK9* is a member of the proprotein convertase subtilisin/kexin family. Mutations in *PCSK9* have been identified in familial autosomal dominant hypercholesterolemia patients, and gain-of-function mutations increase LDL cholesterol concentration [1,2]. The major molecular function of PCSK9 in LDL cholesterol and lipid homeostasis is degradation of the LDL receptor (LDLR), VLDL receptor (VLDLR) and LDLR-related protein 8 (LRP8) [3-5]. In addition, several studies in mice and non-human primates have shown that PCSK9 is involved in HDL metabolism. *Pcsk9* KO male mice on a B6 background fed a chow diet exhibited a 30% reduction in HDL cholesterol concentration [6]. B6 male mice fed a high fat diet and then treated with a *Pcsk9* antisense oligonucleotide inhibitor for 6 weeks showed a 54% reduction in HDL cholesterol concentration [7]. In male cynomolgus macaques, treatment with neutralizing antibodies against PCSK9 reduced HDL cholesterol concentrations for the first seven days of treatment [8]. Despite the accumulating evidence, the molecular mechanism by which PCSK9 regulates HDL cholesterol concentration has not been investigated. Previous studies reported decreased levels of circulating APOE and higher levels of LDLR, VLDLR, and LRP8 by PCSK9 inhibition [4-6]. APOE in lipoproteins acts as a ligand of LDLR family proteins and promotes lipoprotein particle clearance [9,10]. APOE is an efficient cholesterol acceptor in HDL, and the binding of APOE in newly secreted HDL (also called nascent HDL) increases the particle size and cholesterol concentration [11,12]. Thus, PCSK9-mediated regulation of APOE levels in HDL may be a key mechanism that determines HDL cholesterol concentration. In this study, we show that increased LDLR decreases APOE-containing HDL subfractions and HDL cholesterol concentrations in mice. We further demonstrate that, although LDLR plays an important role in PCSK9-mediated regulation of HDL cholesterol concentration, PCSK9 does not entirely rely on LDLR and that PCSK9-mediated regulation of HDL cholesterol concentration relies entirely on the presence of APOE. Finally, we show that, although *Pcsk9* KO reduces HDL cholesterol concentration and cholesterol efflux capacity in serum, there is no significant impact on early atherogenesis.

## Results

### PCSK9-mediated HDL cholesterol regulation is partially sex- and diet-dependent

**Figure 1:**
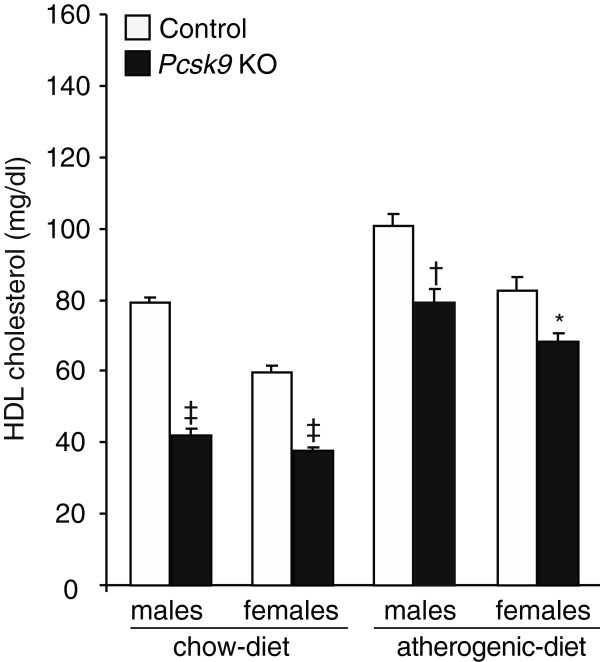
**HDL cholesterol concentrations in control and *Pcsk9 *KO males and females.** Plasma was obtained from male and female mice (n > 10 per genotype) fed a normal chow diet from weaning until 8 weeks of age and then an atherogenic diet until 16 weeks of age. Data represent the mean ± SEM. *P < 0.05, ^†^*P* < 0.001, ^‡^*P* < 0.0001.

To validate the effect of PCSK9 on the regulation of HDL cholesterol concentration, we compared HDL cholesterol concentrations in *Pcsk9* KO and control males and females on a chow diet and an atherogenic diet (Figure 1). Compared to control mice, all *Pcsk9* KO mice had lower HDL cholesterol concentrations. In *Pcsk9* KO males, concentrations were decreased by 47% on a chow diet (KO, 42.1 ± 1.3 mg/dl; control, 79.2 ± 1.9 mg/dl; *P* < 0.0001) and by 21% on an atherogenic diet (KO, 79.2 ± 4.0 mg/dl; control, 100.6 ± 3.5 mg/dl; *P* < 0.001). In *Pcsk9* KO females, concentrations were decreased by 37% on a chow diet (KO, 37.8 ± 0.9 mg/dl; control, 59.7 ± 1.7 mg/dl; *P* < 0.0001) and by 17% on an atherogenic diet (KO, 68.4 ± 2.1 mg/dl; control, 82.8 ± 3.8 mg/dl; *P* < 0.05). Also, the differences in HDL cholesterol concentrations between *Pcsk9* KO mice and controls were smaller in mice fed the atherogenic diet than in mice fed the chow diet. These results indicate that PCSK9-mediated regulation of HDL cholesterol concentrations is partially dependent on sex and diet.

### APOE in non-HDL depleted serum (NHDS) is significantly reduced in *Pcsk9* KO mice

To investigate how PCSK9 regulates HDL cholesterol concentration, we examined whether APOE composition and distribution in HDL were affected in *Pcsk9* KO mice. We performed mass spectrometry using the NHDS of 8-week-old *Pcsk9* KO and control mice. The NHDS was separated on a non-denaturing 4-30% polyacrylamide gel that exhibited 11 bands after staining with Coomassie Brilliant Blue R-250. Each band was excised as a 1-mm-wide slice for mass spectrometry to identify apolipoproteins; results were compared between *Pcsk9* KO and control mice. As expected, APOA1, but not APOB, was present in all bands (data not shown). The presence of APOA1 indicated that the bands contained HDL, while the absence of APOB indicated that APOB-containing lipoproteins were effectively removed by the precipitation method. In *Pcsk9* KO mice, APOE was not found in any bands, while in control mice, APOE was found in the five bands with a molecular weights above 272 kDa (See Additional file 1: Table S1). We next tested whether the absence of APOE results from decreased APOE production. Compared to wild-type controls, *Pcsk9* KO mice showed no decrease in *Apoe* expression and APOE protein level in the liver (See Additional file 2: Figure S1) and in the peritoneal macrophages (data not shown) where APOE is mainly produced. Combined, these results suggest that the reduced APOE level in *Pcsk9* KO NHDS is not due to decreased APOE production.

### APOE-containing HDL subfractions are decreased in *Pcsk9* KO mice

**Figure 2:**
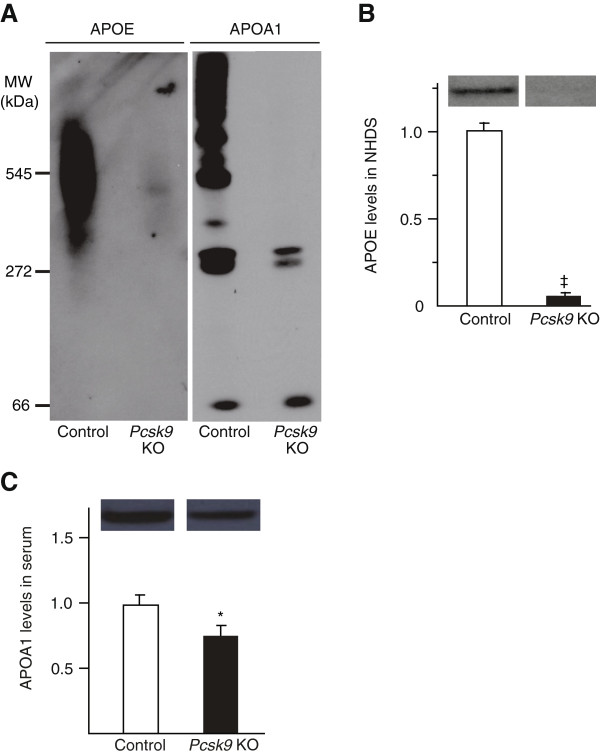
**APOE and APOA1 levels in HDL subfractions in control and *Pcsk9 *KO mice.** NHDS obtained from 5 males per genotype was electrophoresed on non-denaturing gels and blotted using antibodies against APOE and APOA1 **(A)**. Fifteen μg of proteins were separated by SDS-PAGE and blotted using antibody against either APOE or APOA1. The relative intensity of the APOE **(B)** and APOA1 **(C)** bands is shown. Data represent the mean ± SEM. * *P* < 0.05, ^‡^*P* < 0.0001.

We next investigated whether APOE reduction in the NHDS of *Pcsk9* KO was due to reduced APOE-containing HDL subfractions. APOA1 is a hallmark of HDL, and a reduction of APOA1 levels in serum indicates a decrease in HDL levels. We ran NHDS of *Pcsk9* KO and control mice on 4-30% non-denaturing polyacrylamide gels and performed western blotting for both APOE and APOA1 (Figure 2A). APOE was detected in HDL subfractions exhibiting an apparent molecular weight larger than 272 kDa. Both APOE and APOA1 levels were severely decreased in the *Pcsk9* KO HDL subfractions compared to those of control mice. To quantify the levels of APOE and APOA1, we performed SDS-PAGE and western blots. The results showed 80–90% reduced APOE in *Pcsk9* KO NHDS (Figure 2B). The APOA1 level was 20–30% reduced in *Pcsk9* KO serum (Figure 2C) and 30% reduced in pooled *Pcsk9* KO NHDS compared to pooled control NHDS (n = 3 per genotype, data not shown). These results suggest that decreased APOE levels in *Pcsk9* KO NHDS are due to both a decrease in HDL particle numbers (−25% APOA1) and a smaller fraction of APOE bound to HDL particles. In the 66-kDa HDL subfraction that does not contain APOE, APOA1 band intensity was similar between *Pcsk9* KO mice and controls. Combined, our results suggest that *Pcsk9* inactivation specifically reduces APOE-containing HDL subfractions.

### Increased LDLR leads to decreased levels of HDL cholesterol concentration and APOE-containing HDL subfractions

**Figure 3:**
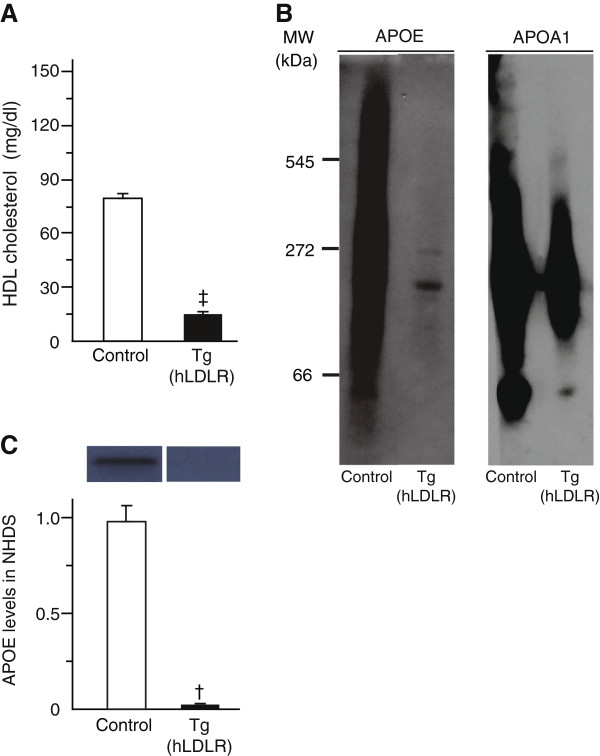
**HDL cholesterol concentrations in serum, APOE and APOA1 levels in HDL subfractions in control and Tg(hLDLR) mice.** Serum and NHDS were obtained from serum of 8-week-old males (n = 5 per genotype). HDL cholesterol concentration was measured in control and Tg(hLDLR) mice **(A)**. HDL subfractions were electrophoresed in a non-denaturing gel and blotted using antibodies for APOE and APOA1 **(B)**. Fifteen μg of proteins of NHDS were separated by SDS-PAGE and blotted using an APOE antibody. The relative band intensity of APOE is shown **(C)**. Data represent the mean ± SEM. ^†^*P* < 0.001.

We investigated the molecular mechanism behind the reduced level of APOE-containing HDL in *Pcsk9* KO mice. We hypothesized that LDLR is an important component of the mechanism because LDLR is a receptor that binds APOE in lipoproteins [13] and the LDLR level is increased in *Pcsk9* KO mice [6]. We thus analyzed HDL cholesterol concentration in Tg(hLDLR) mice that overexpress LDLR (Figure 3A). Similar to *Pcsk9* KO mice, Tg(hLDLR) mice exhibited 82% lower HDL cholesterol concentrations compared to control mice (Tg, 14.3 ± 1.3 mg/dl; control, 79.1 ± 2.3 mg/dl; *P* < 0.0001). They exhibited lower APOE and APOA1 levels in HDL subfractions larger than the molecular weight of 272 kDa (Figure 3B). Distribution of APOA1 in NHDS of Tg(hLDLR) controls was different from that of *Pcsk9* KO controls, which is likely due to the mixed genetic background (SJL/J and C57BL/6 J) of the Tg(hLDLR) controls vs. the C57BL/6 J background of the *Pcsk9* KO controls. The drop in APOE (80–90%) (Figure 3C) and APOA1 (53%) (n = 3 per genotype, data not shown) was further confirmed by western blot analysis of NHDS. These results confirmed that LDLR overexpression in mice reduces the concentrations of HDL cholesterol and APOE-containing HDL.

### PCSK9 regulates HDL cholesterol concentration through LDLR and APOE

**Figure 4:**
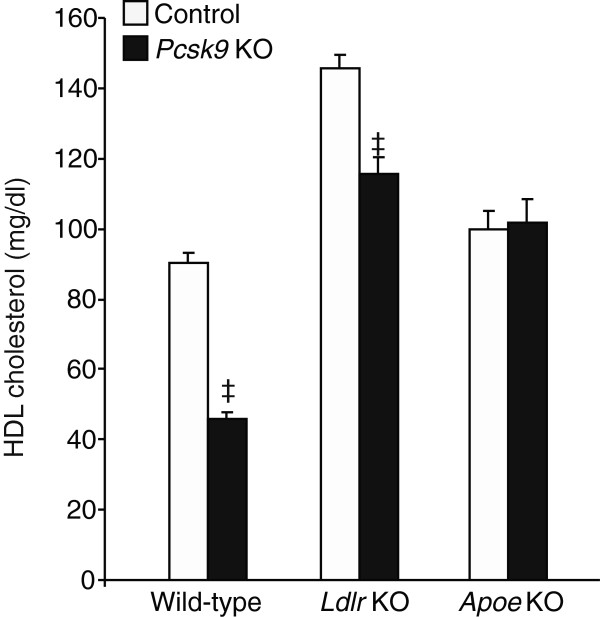
**Effect of PCSK9 on HDL cholesterol concentration in mice having a wild-type control, *Ldlr *KO or *Apoe *KO background.** Fasting plasma samples were obtained from 3-month-old mice (n = 5 per genotype) fed a normal chow diet. HDL cholesterol concentrations were measured in mice that expressed normal endogenous PCSK9 (white control bars) or were deficient in PCSK9 (black bars). Data represent the mean ± SEM. ^‡^*P* < 0.0001.

We verified whether LDLR was the unique receptor for the PCSK9-mediated HDL regulation by measuring HDL cholesterol concentrations in *Ldlr* KO, *Pcsk9*/*Ldlr* double-KO and wild-type control mice (Figure 4). HDL cholesterol concentrations in *Ldlr* KO were 1.6-fold higher than in wild-type control mice (145.6 ± 4.1 mg/dl vs 90.3 ± 3.4 mg/dl, *P* < 0.001). However, the absence of PCSK9 combined with the absence of LDLR resulted in a 1.2-fold induction of HDL cholesterol levels (115.5 ± 4.5 mg/dl vs 90.3 ± 3.4 mg/dl, *P* < 0.001), indicating that PCSK9 does not completely rely on an LDLR-dependent mechanism to regulate HDL cholesterol concentration.

We also tested whether APOE was required for PCSK9-mediated HDL regulation. HDL cholesterol concentrations were measured in *Apoe* KO and *Pcsk9/Apoe* double-KO males and were found similar (101.6 ± 7.0 mg/dl vs 99.9 ± 4.5 mg/dl). These results indicate that PCSK9-mediated HDL regulation — either LDLR-dependent or LDLR-independent — completely relies on APOE.

**Figure 5:**
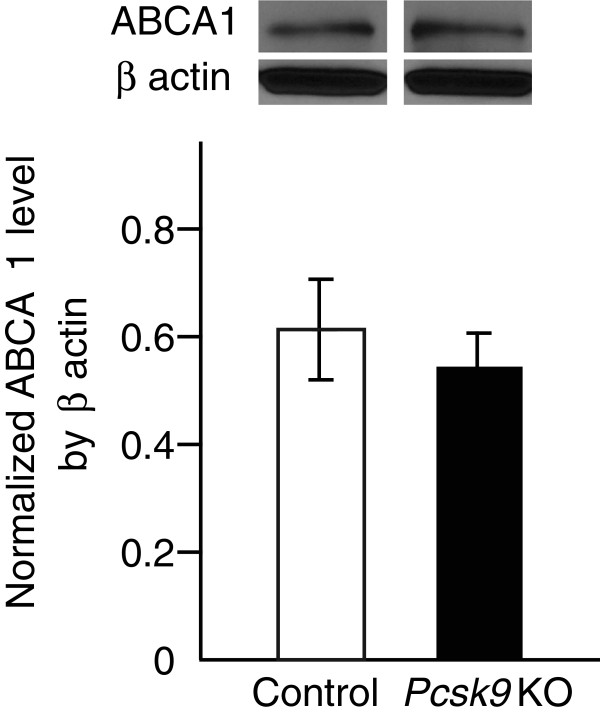
**ABCA1 level in livers from control and *Pcsk9 *KO mice.** The whole liver was obtained from 8-week-old control and *Pcsk9* KO males (n = 5 per genotype). One hundred μg of proteins were separated by SDS-PAGE and blotted using antibody against ABCA1. ABCA1 level was normalized by β actin. Data represent the mean ± SEM.

Scavenger receptors type 1 (SR-B1) is an important factor for HDL clearances in mice [14,15], however two recent publications reported no relationship between SR-B1 and PCSK9. Rashid et al., stated that SR-B1 level is not changed in *Pcsk9* knockout mouse livers [6] and Lalanne et. al., showed that adenoviral PCSK9 transduction in C57BL/6 mice does not change SR-B1 level in livers [16]. In addition to SR-B1, ATP-binding cassette transporter A1 (ABCA1) plays an important role for maintaining circulating HDL cholesterol concentrations through APOA1 and APOE-dependent efflux [17], we tested whether ABCA1 level is changed in *Pcsk9* KO mouse livers and found no difference in ABCA1 level between WT controls and *Pcsk9* KO mouse livers (Figure 5). Taken together, no change of SR-B1 and ABCA1 levels in *Pcsk9* KO mouse livers support that reduced HDL cholesterol concentration in *Pcsk9* KO mouse serum is largely through the clearance of APOE-containing HDL subfractions.

### Effects of *Pcsk9* inactivation on the cholesterol efflux capacity of serum and atherosclerotic fatty streak volume in aortic sinus

**Figure 6:**
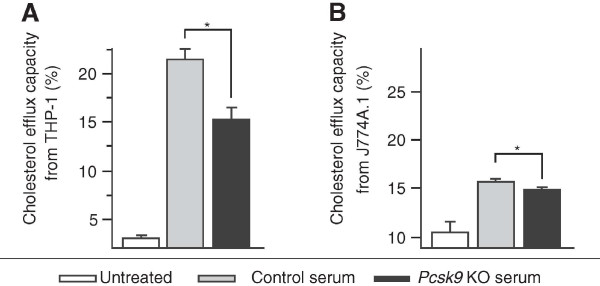
**Cholesterol efflux capacity in control and *Pcsk9 *KO serum.** Cholesterol efflux capacity (%) was calculated as F-cholesterol efflux (%) = F-cholesterol in medium / (F-cholesterol in medium + F-cholesterol in cells) × 100. Cholesterol efflux was induced by 5 μl serum incubation in triplicate (n = 5 per genotype) in THP-1 for 1 hour **(A)** and J774A.1 macrophage foam cells for 48 hours **(B)**. Data represent the mean ± SEM. *P < 0.05.

**Figure 7:**
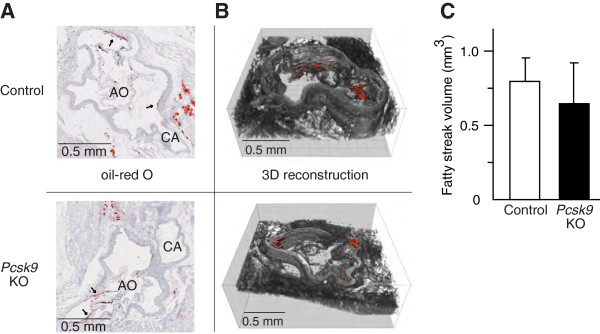
**Atherosclerotic fatty streak volume in control and *Pcsk9 *KO mice.** Control and *Pcsk9* KO males (34-week-old; n = 5 per genotype) were fed an atherogenic diet for 10 weeks. Aortic sections were stained with oil-red O **(A)** and fatty streaks are indicated by arrows **(A)**, 3D image was reconstructed **(B)** and the fatty streak volume (mm^3^) quantified in 240 μm of aortic sinus **(C)**. Data represent the mean ± SEM.

To test whether *Pcsk9* inactivation affects cholesterol efflux capacity of serum, macrophage foam cell formation was induced and cells were treated with mouse serum as previously described [18]. The amount of a fluorescent cholesterol mimic present in cells and released in the culture media was then measured. Two lines of macrophages were tested, the human THP-1 and mouse J774A.1 cells. In THP-1 cells, incubation with *Pcsk9* KO serum resulted in significant lower cholesterol efflux capacity than with control serum (Figure 6A). Slower and very small reduction of cholesterol efflux in J774.A1 cells than in THP-1 cells is likely due to deficient APOE secretion (Figure 6B) [19]. As inefficient cholesterol removal from lesions increases the atherosclerotic burden [20], we tested whether early atherogenesis is increased by measuring fatty streak volume in *Pcsk9* KO mice. We compared atherosclerotic fatty streak volume in 34-week-old *Pcsk9* KO and control males after a 10-week atherogenic diet. The fatty streaks in the same 240-μm region of the aortic sinus were visualized by oil red O staining (Figure 7A) and then digitalized and reconstructed in 3D images (Figure 7B). Compared to controls, the fatty streak volume in *Pcsk9* KO mice was similar (Figure 7C) while HDL cholesterol concentration was 24% lower (KO, 73.5 ± 7.5 mg/dl; control, 97.1 ± 5.2 mg/dl; *P* < 0.05). Altogether, these results suggest that although the cholesterol efflux capacity of *Pcsk9* KO serum from THP-1 macrophage foam cells is reduced, *Pcsk9* inactivation does not have a measurable impact on early atherogenesis under our experimental conditions.

## Discussion

In this study, we revealed the molecular mechanism by which PCSK9 controls HDL cholesterol concentration by regulating the APOE-containing HDL. We show that APOE and APOA1 in the same size of HDL subfractions were reduced in *Pcsk9* KO mice. Combined with the result showing no impact on smaller 66-kDa sized HDL level, our data suggest that PCSK9 specifically controls APOE-containing HDL.

Because APOE-containing lipoproteins are mainly cleared via LDLR [10,21] and *Pcsk9* KO mice exhibiting 2- to 3-fold higher levels of LDLR in the liver [6,22], we hypothesized that rapid clearance of APOE-containing HDL via LDLR might be the major cause of decreased HDL cholesterol concentration in *Pcsk9* KO mice. This hypothesis was reinforced by the analysis of Tg(hLDLR) mice that overexpress LDLR. They exhibit very low levels of APOE-containing HDL, and their HDL cholesterol concentrations are reduced by 82% (versus about 47% in *Pcsk9* KO mice). Because APOE is known to promote cholesterol loading on HDL [11], this dramatic 82% reduction likely reflects a lower HDL cholesterol concentration due to the faster clearance of the APOE-containing HDL and the poor cholesterol concentration in the remaining smaller lipid-free or lipid-poor HDL subfraction. Our data clearly points at the key role of LDLR in the clearance of HDL.

We also show that, although LDLR plays an important role in PCSK9-mediated HDL cholesterol regulation, it is not a unique factor implicated in this process. HDL cholesterol concentrations in *Pcsk9*/*Ldlr* double-KO mice were 21% lower than in *Ldlr* KO mice, revealing an LDLR-independent effect of PCSK9. No change of ABCA1 and SR-B1 levels in *Pcsk9* KO mouse livers support that reduced HDL cholesterol concentration in *Pcsk9* KO mouse serum is largely through the clearance of APOE-containing HDL subfractions. A possible LDLR-independent mechanism may be based on APOE binding to the VLDLR, as APOE is an efficient ligand of VLDLR. VLDLR is targeted for degradation by PCSK9 in HEK293 and NIH 3 T3 cell lines [4], and cell surface VLDLR levels were increased in the adipose tissue of *Pcsk9* KO mice [5]. We finally show that HDL cholesterol concentrations are similar between *Apoe* KO and *Pcsk9*/*Apoe* double-KO males, suggesting that the PCSK9-mediated HDL cholesterol regulation is dependent on the presence of APOE and that the role of APOE entirely depends on its ability to mediate the binding of HDL to LDLR or VLDLR.

Therapeutic inhibition of PCSK9 is a promising pro-atherogenic LDL cholesterol-lowering treatment. Co-treatment of PCSK9 inhibitors with drugs that suppress cholesterol synthesis is even more effective in reducing LDL cholesterol in hypercholesterolemia patients [23-25]. Inconsistent with recent clinical trials, studies in laboratory animals show that PCSK9 inhibition reduces HDL cholesterol concentration. The inconsistency might be due to different dosage of PCSK9 inhibition in different studies. Or, a reduction in HDL cholesterol concentration by PCSK9 inhibition might be restricted to species such as the mouse in which APOE-containing HDL level is elevated, compared to human [26-28].

Independently of the level of HDL cholesterol concentration, cholesterol efflux capacity is associated with atherosclerotic plaque formation in the coronary arteries [20]. We found that *Pcsk9* KO leads to a reduction in cholesterol efflux capacity of serum from THP-1 macrophage foam cells, but there was no significant impact on atherogenic-diet induced fatty streak volume in aortic sinus. Similar observation was made in a recent study showing no increase of atherosclerotic lesion size in the aortas of *Pcsk9/Ldlr* double-KO and *Pcsk9/Apoe* double-KO mice. We speculate that this might be because *Pcsk9* inactivation reduces pro-atherogenic LDL level in the circulation and also reduces the accumulation of esterified cholesterol in the aortas [29].

## Conclusions

In mice, PCSK9 controls circulating cholesterol concentrations by regulating both LDL and HDL levels through LDLR. Our data suggest that the regulation of HDL by PCSK9 is mainly through LDLR-mediated APOE-containing HDL clearance and that other targets of PCSK9 might be involved in the process [5,30]. Our data validate that reduced HDL cholesterol concentration and cholesterol efflux capacity in serum by *Pcsk9* inactivation do not have significant impact on the early stage of atherosclerosis development.

## Methods

### Mice, husbandry and diet

Eight different strains of mice plus two types of control mice were used in this study (Table 1). Mice were maintained in a pathogen-free and climate-controlled facility with a 12-hour light/dark cycle and fed *ad libitum* throughout the experiment. *Pcsk9* KO, Tg(hLDLR) and their control mice were fed a normal chow diet containing 6% fat (5 K52 LabDiet®, PMI Nutrition International, St. Louis, MO). At eight weeks of age, a group of *Pcsk9* KO and control mice were switched to an atherogenic diet until 16 weeks of age (18.5% dietary fat, 1.9% corn oil, 50% sucrose, 4.1% cellulose, 20% casein, 1% cholesterol, 0.5% cholic acid, 5% mineral mix, 1% vitamin mix, 0.3% DL-methione, 0.13% DL-a-tochopherol, 1% choline chloride [31]). Another group of *Pcsk9* KO and control mice were fed the same atherogenic diet between 24 and 34 weeks of age. *Pcsk9/Ldlr* double-KO, *Pcsk9/Apoe* double-KO, *Pcsk9* KO IRCM, *Ldlr* KO, *Apoe* KO, wild-type control mice were fed a regular diet containing 6% fat (2018 Teklad Global) diet until three months of age [29]. Experiments were approved by the Institutional Animal Care and Use Committee of The Jackson Laboratory, Bar Harbor, ME and by the Animal Care Committee of The Institute de Recherches Cliniques de Montréal (IRCM), Montreal, QC.

**Table 1:** Descriptions of mice used in this study

**Mouse strain**		**Control mice**
**Abbreviation**	**Full name, description**	
*Apoa1* KO	B6;129P2-*Apoa1*^*tm1Unc*^/J (JAX® 002055)	C57BL/6 J
*Apoe* KO	B6.129P2-*Apoe*^*tm1Unc*^/J (JAX® 002052) backcrossed to B6	C57BL/6 J
Tg(hLDLR)	Hemizygous male offspring of hemizygous B6;SJL-Tg(Mt1-LDLR)93-4Reh/AgnJ (JAX® #008850) males mated with B6SJLF1/J (JAX® #100012) females	Wild-type male littermates of Tg(hLDLR)
*Ldlr* KO	B6.129S7-*Ldlr*^*tm1Her*^/J (JAX® 002207) backcrossed to B6	C57BL/6 J
*Pcsk9* KO	B6;129S6-*Pcsk9*^*tm1Jdh*^/J (JAX® 005993) backcrossed to B6	C57BL/6 J
*Pcsk9* KO IRCM	B6 mice that lack the *Pcsk9* proximal promoter and exon 1 region	C57BL/6 J
*Pcsk9/Apoe* double-KO	*Apoe* KO mice crossed to *Pcsk9* KO IRCM mice	C57BL/6 J
*Pcsk9/Ldlr* double-KO	*Ldlr* KO mice crossed to *Pcsk9* KO IRCM mice	C57BL/6 J

C57BL/6 J is JAX® 000664.

### HDL cholesterol measurement

At 8 and 16 weeks of age, mice were fasted from 07:00 am to 11:00 am and then retro-orbitally bled; 100–150 μl of blood was collected in a 1.5 ml tube for serum and in a 1.5 ml tube containing 5 μl of 200 μM ethylenediaminetetraacetic acid (EDTA) for plasma. Serum or plasma was isolated by centrifugation at 15,000 rpm for five minutes at room temperature within two hours of the bleed. Collected supernatant was stored at −20°C until the HDL cholesterol concentration was measured by the HDLD assay, using an enzymatic reagent kit (Beckman Coulter Inc., Palo Alto, CA) on a Beckman Synchron DXC (Beckman Coulter Inc., Palo Alto, CA). The HDL method used for HDLD assay was validated in mice and was used in our previous publications [32,33]. At three months of age, plasma samples were obtained from *Pcsk9/Ldlr* double-KO, *Pcsk9/Apoe* double-KO, *Pcsk9* KO IRCM, *Ldlr* KO, *Apoe* KO, and B6 males as previously described [29]. At 34 weeks of age, non-fasting serum HDL cholesterol concentration was measured in the group of *Pcsk9* KO and control mice that were fed an atherogenic diet for 10 weeks [34].

### Preparation of non-HDL depleted serum (NHDS) and identification of APOE and APOA1

At eight weeks of age, mice were singly housed for four days. Blood was collected and serum was isolated as described above. The NHDS was collected using one-tenth volume of the chemical precipitation reagent containing dextran sulfate (10 g/L), magnesium ions (500 mM), and non-reactive ingredients with sodium azide (0.1%), where the binding of the reagent to serum precipitates the LDL and VLDL [35]. After precipitation, the NHDS was collected and then total protein concentration (μg/μl) was determined using a Bradford assay (Sigma Life Sciences, St. Louis, MO). Fifteen μg of proteins in NHDS were run on a 4-30% non-denaturing polyacrylamide gradient gel [36], and proteins bands were visualized by Coomassie Brilliant Blue R-250. The molecular weight of the different bands was determined using a molecular weight kit (14–500 kDa) (Sigma Life Sciences, St. Louis, MO) according to the manufacturer’s instructions. To identify proteins, Coomassie Brilliant Blue R-250-stained bands were cut into 1 mm^3^ cubes, proteins in each band were digested in trypsin solution, and the tryptic peptides were subjected to LC-MS/MS. Mass spectrometry data were collected and MS spectra were searched against an IPI mouse protein sequence database (version 3.75) using SEQUEST (Bioworks software, v3.3.1; Thermo Electron) [37].

### Western blot

Proteins in whole liver were prepared in protein extraction buffer (T-PER reagent, Roche, Indianapolis, IN) and a protease inhibitors cocktail tablet (Roche, Indianapolis, IN). Fifteen μg of proteins in NHDS were electrophoresed by SDS-PAGE or native-PAGE, and western blotting was performed using antibodies for APOE (ab20874, polyclonal rabbit primary 1/1,000, Abcam, Cambridge, MA), APOA1 (ab20453, polyclonal rabbit primary 1/1,000, Abcam, Cambridge, MA), ABCA1 (MAB10005, monoclonal mouse primary 1/750, EMD Millipore, Temecula, CA), β actin (ab8227, polyclonal rabbit primary 1/25,000, Abcam, Cambridge, MA) and secondary antibodies for anti-rabbit IgG (7074S, HRP-linked secondary 1/5,000, Cell Signaling Technology Inc., Danvers, MA) and for anti-mouse IgG (AP308P, HRP-linked secondary 1/5,000, EMD Millipore, Temecula, CA). Protein levels were calculated by the protein band intensity that was obtained using ImageJ 1.44o (National Institute of Health, Bethesda, MD).

### Quantitative PCR of *Apoe*

Total RNA in liver was extracted using a Trizol Plus RNA Purification kit (Invitrogen Life Technologies, Grand Island, NY). Complementary DNA (cDNA) was synthesized using an Omniscript RT kit (Qiagen, Valencia, CA) and used for qPCR with SYBR green (Applied Biosystems, Inc., Foster City, CA) and *Apoe* primers (Forward: 5′ AACCGCTTCTGGGATTACCTG 3′ and Reverse: 5′ TCAGTTCTTGTGTGACTTGGGA 3′ from Primerdesign Ltd., Southampton, UK). The *Apoe* expression level was normalized by *Gapdh* (Forward: 5′ TGGTGAAGGTCGGTGTGAAC 3′ and Reverse: 5′ CAATGAAGGGGTCGTTGATGG 3′ from Primerdesign Ltd., Southampton, UK). Relative expression differences were obtained using LinRegPCR (v11.0) [38] and the Relative Expression Software Tool (REST©) [39].

### *In vitro* assessment of cholesterol efflux capacity of serum from macrophage foam cells

THP-1 and J774A.1 cells were purchased from American Type Culture Collection (ATCC, Manassas, VA) and cultured in ATCC-formulated DMEM based media and RPMI-1640, respectively, at 5% CO_2_ atmosphere at 37°C. Low passage cells (p0–2) were used throughout the experiment. Both cell types were treated with 10 nM of phorbol myristate acetate (PMA) to differentiate into macrophages. As described previously [18], the differentiated macrophages were treated with 10 μM of fluorescent cholesterol mimic (F-cholesterol) [40] and 50 μg/ml oxidized LDL (Biomedical Technologies, Inc., Stoughton, MA) to induce macrophage foam cell formation. The macrophage foam cells were used to perform a cell-based, high-throughput screening assay for cholesterol efflux. Five μl of serum was added in 100 μl of cell culture medium (n = 5 per strain) in triplicate and left for 48 hours for J774A.1 macrophage foam cells and 1 hour for THP-1 macrophage foam cells. Fluorescent cholesterol mimic (F-cholesterol) in cells and media was measured in separate wells at 485/535 excitation/emission wavelengths. Cholesterol efflux capacity (%) was calculated as F-cholesterol efflux (%) = F-cholesterol in medium ∕ (F-cholesterol in medium + F-cholesterol in cells) × 100.

### Histological assessment of atherosclerotic fatty streak volume

Fatty streak volume was assessed as previously described [41] with minor modifications. In brief, 24-week-old *Pcsk9* KO and control males were fed an atherogenic diet for 10 weeks. Supplementation of 0.5% cholic acid in the diet increases intestinal cholesterol absorption, which then accelerates atherosclerosis development. Hearts were collected, embedded in optimal cutting temperature (OCT) compound, sliced in 10-μm thick sections, placed on glass slides and fixed in 10% formalin. Lipids and esterified cholesterol were stained with oil red O and counter-stained with Mayer’s hematoxylin [42]. Images of slides were digitalized using a Nanozoomer (Hamamatsu, Bridgewater, NJ). To identify the identical histological region for all animals, the area in the aortic sinus where the coronary artery and ascending aorta join was used as a landmark. For each animal, individual digitalized images of 12 sections above and 12 sections below the landmark were loaded into FIJI (NIH, Bethesda, WD) and saved as Z-stacks. These Z-stacks were loaded into AutoAligner (Bitplane AG, Zürich, Switzerland) to align sections and then opened in Imaris (Bitplane AG, Zürich, Switzerland) to reconstruct 3-dimensional images and calculate the average volume of atherosclerotic fatty streak (mm^3^).

### Statistics

All data represent the mean ± SEM from the number of animals of each group. For comparisons of two groups, levels of significance were calculated with the two-sample t-test using JMP9 (SAS Institute, Inc., Cary, NC).

## Competing interests

The author declare no conflicts of interest, state that the manuscript has not been published or submitted elsewhere, state that the work complies with the ethical policies of the journal and state that the work has been conducted under internationally accepted ethical standards after relevant ethical review.

## Authors’ contributions

SC, as the first author, provided substantial contribution in this study; conception; design; data acquisition, analysis and interpretation; manuscript preparation. AA participated in histological assessment of fatty streak volume and provided significant intellectual content. US carried out the non-denaturing gel electrophoresis and prepared samples for mass spectrometry. BD carried out mass spectrometry and analyzed apolipoprotein profile. B.R.P established a protocol of cholesterol efflux capacity assay and provided fluorescently labeled cholesterol mimic. AP provided plasma samples of C57BL/6, *Ldlr* KO, *Apoe* KO, *Pcsk9* KO, *Pcsk9/Ldlr double-KO*, *Pcsk9/Apoe* double-KO mice for HDL cholesterol measurement and significant intellectual content. RK, as a Ph.D. mentor of SC and corresponding author of this study, supervised the entire process of research and publication. All authors read and approved the final manuscript.

## Supplementary Material

Additional file 1: Table S1 APOE distribution in HDL subfractions of contro l an d *Pcsk9* KO mice. Click here for file

Additional file 2: Figure S1 APOE production in livers from control and *Pcsk9* KO mice. Click here for file
