# Supplementary material for: Decreased APOE-containing HDL subfractions and cholesterol efflux capacity of serum in mice lacking Pcsk9
Source: Lipids Health Dis. 2013 Jul 24;12:112. doi: 10.1186/1476-511X-12-112 (PMC3751695; doi:10.1186/1476-511X-12-112)
Supplement: Additional file 1: Table S1 — APOE distribution in HDL subfractions of contro l an d Pcsk9 KO mice. [file 1476-511X-12-112-S1.pdf]

**Additional file 1.**

**Supplemental Table 1.** APOE distribution in HDL subfractions of control and *Pcsk9* KO mice.

| Molecular Weight (kDa) | HDL Subfractions | Control | *Pcsk9* KO |
|---|---|---|---|
| | 1 | X | - |
| | 2 | X | - |
| 545 | 3 | X | - |
| | 4 | X | - |
| | 5 | X | - |
| 272 | 6 | - | - |
| | 7 | - | - |
| | 8 | - | - |
| 132 | 9 | - | - |
| | 10 | - | - |
| 66 | 11 | - | - |

NHDS was obtained from pooled serum of 8-week-old control and *Pcsk9* KO males (n = 2 per strain). Ten µl of NHDS were electrophoresed using a non-denaturing gel, and HDL subfractions were visualized by staining with Coomassie Brilliant Blue R-250. APOE in each HDL subfraction was identified by mass spectrometry, and its distribution was compared between control and *Pcsk9* KO mice. The presence of APOE is indicated as (X) and the absence as (-).
