# Supplementary material for: Decreased APOE-containing HDL subfractions and cholesterol efflux capacity of serum in mice lacking Pcsk9
Source: Lipids Health Dis. 2013 Jul 24;12:112. doi: 10.1186/1476-511X-12-112 (PMC3751695; doi:10.1186/1476-511X-12-112)
Supplement: Additional file 2: Figure S1 — APOE production in livers from control and Pcsk9 KO mice. [file 1476-511X-12-112-S2.pdf]

Additional file 2

Supplemental Figure 1 . APOE production in livers from control and *Pcsk9* KO mice.

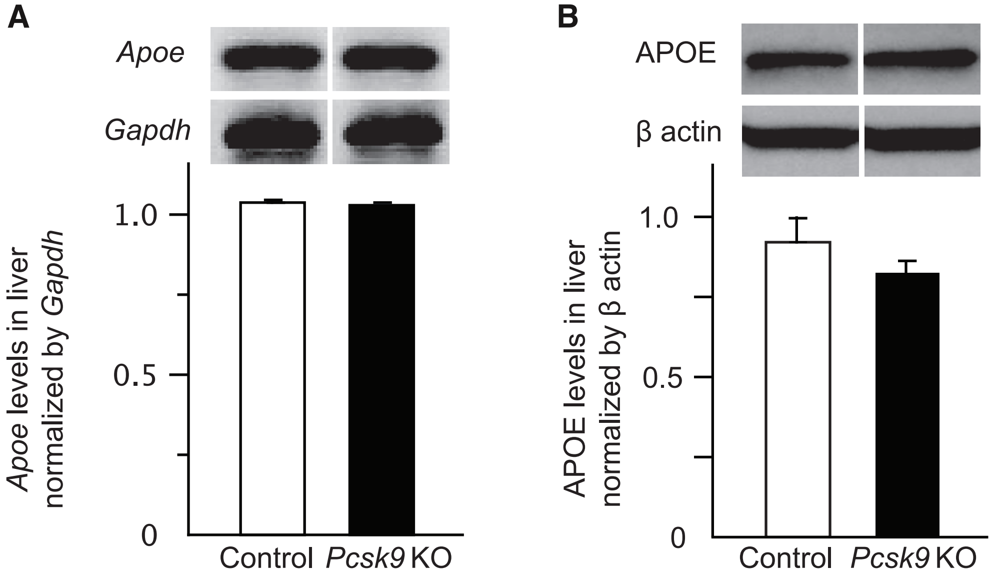

The whole liver was obtained from 8-week-old control and *Pcsk9* KO males (n = 5 per strain). The liver was pulverized and divided into two vials, one for RNA and the other for protein extraction. *Apoe* expression levels were normalized by *Gapdh* (A) and APOE protein levels were normalized by β actin (B). Data represent the mean ± SEM from the number of animals of each strain.
